# Negative Life Events and Attempted Suicide in Rural China

**DOI:** 10.1371/journal.pone.0116634

**Published:** 2015-01-22

**Authors:** Wen-Chao Zhang, Cun-Xian Jia, Ji-Yu Zhang, Lin-Lin Wang, Xian-Chen Liu

**Affiliations:** 1 Department of Epidemiology and Health Statistics, Shandong University School of Public Health, Jinan, China; 2 Shandong University Center for Suicide Prevention Research, Jinan, China; 3 Shandong Center for Disease Prevention and Control, Jinan, China; 4 Ji’nan Shizhongqu Center for Disease Prevention and Control, Jinan, China; 5 University of Tennessee Health Science Center, Memphis, Tennessee, United States of America; Alberta Provincial Laboratory for Public Health/ University of Alberta, CANADA

## Abstract

**Objective:**

This study aimed to examine the association between negative life events (NLEs) and attempted suicide in rural China.

**Methods:**

Six rural counties were selected from disease surveillance points in Shandong province, China. A total of 409 suicide attempters in rural areas between October 1, 2009, and March 31, 2011, and an equal number of matched controls were interviewed. We compared negative life events experienced within 1 month, 1–3 months, 3–6months, and 6–2 months prior to attempted suicide for cases and prior to interview for controls. We used multivariate logistic regression to examine the association between NLEs and attempted suicide.

**Results:**

Suicide attempters experienced more NLEs within the last year prior to suicide attempt than controls prior to interview (83.1% vs. 33.5%). There was a significant dose-response relationship between NLEs experienced within the last year and increased risk of attempted suicide. Timing of NLEs analysis showed that NLEs experienced in the last month and 6–12 months prior to suicide attempt were significantly associated with elevated risk of attempted suicide, even after adjusting for mental disorders and demographic factors. Of NLEs, quarrelling with spouse, quarrelling with other family members, conflicting with friends or neighbors, family financial difficulty, and serious illness were independently related to attempted suicide.

**Conclusion:**

NLEs are significantly associated with increased risk for attempted suicide in rural China. Stress management and intervention may be important to prevent suicidal behavior in rural China.

## Introduction

Suicide is an important global public health concern that claims approximately 1 million premature deaths every year, or 1 suicidal death occurs every 40 seconds [1]. While we already know that suicide patterns in China are different from those in the West, for example, suicide rates are higher in rural areas than in urban areas, especially in rural females [2], we should know more about risk factors that contribute to high suicide risk in rural China.

Given that suicide attempts are one of the most significant predictors of suicide, a meta-analysis estimated that the risk of completed suicide among individuals who attempted suicide is 38–40 times higher than that of the general population [3]. Thus our study aims to understand risk factors for attempted suicide, which are of great value in suicide prevention.

Suicidal risk is multifactorial, spanning from psychosocial, cultural, behavioral, and biological factors [4, 5]. Negative life events (NLEs), commonly defined as objective occurrences of sufficient magnitude to cause negative or adverse life pattern changes for most individuals who experienced them [6], are one of these risk factors. NLEs have been reported to be significantly associated with suicidal behaviors among youth [7], and the risk increased with the frequency of experienced events [8]. The association was also found between NLEs and committed suicide in a case-control study in China [9]. Previous studies have also examined the type of NLEs related to suicidal behavior and found that interpersonal problems (especially emotional issues), financial concerns, unemployment and physical illness were the most common events experienced by suicide attempters [9, 10].

Since these studies of NLEs and suicidal behavior have been conducted in western populations, the findings cannot be generalized to rural Chinese populations. First of all, there are great differences in social, economic and cultural structure between China and the West. Additionally, very few of the western population studies are focused on rural population. Furthermore, the timing of NLEs prior to suicidal behavior may have different impact on suicidal risk. Currently, we are not aware of any studies that examined the timing effects of NLEs on suicide risk.

In this study of suicide attempters in rural China, we aimed: (1) to compare the frequency and type of NLEs between suicide attempters and matched controls; (2) to examine the association between NLEs and attempted suicide after controlling for potential confounders; and (3) to explore the relationship between the timing of NLEs and risk of attempted suicide.

## Methods

### Subjects

Six rural counties (Junan, Lijin, Ningyang, Penglai, Tengzhou, and Zoucheng), were selected from the disease surveillance points (DSPs) to obtain a representative sample of suicide attempters in rural Shandong. Shandong, located in the middle of China’s east coast, is the second largest province by population in China, with 94.17 million people. Approximately 52% of the Shandong’s population resides in rural areas [11]. Shandong is mostly a typical Chinese province in population structure, social pattern and cultural features; however, traditional Confucianism has more profound influences in Shandong. The resulting society is characterized by rigid social hierarchy based on age, gender, and social class [12]. Confucianism defines most kinds of interpersonal relationship vertically and strongly emphasizes personal duties and social goals [13], failure to comply with these rules and in performing one’s role can cause great personal frustrations. Thus studies have been conducted to determine Confucian ethics’ association with suicide. The findings have yielded mixed results. While the Confucian ethic of filial piety has been reported to be negatively associated with suicide, female subordination has been found to be positively associated with suicide [14]. The Confucian ethic of harmony may moderate the impact of negative life events on suicide [14].

This study employed a 1:1 matched case-control design. Cases were consecutive suicide attempters admitted to the emergency department of county-level general hospitals in the 6 counties between October 1, 2009, and March 31, 2011. The local county-level Centers for Disease Prevention and Control (CDCs) collected and reported the hospitalization information of 1070 suicide attempters. Of the reported 1070 attempters, 617 cases were not followed up due to incorrect names or address changes, 44 refused to participate in the study, and 409 were included for the study. A total of 661 cases were not interviewed including 218 men and 443 women. No significant differences existed in mean age (43.90±13.32 vs. 42.85±16.42; *t* = 1.09, *P* = 0.28) and gender distribution (male% = 32.3% vs 33.0%; *χ* ^2^ = 0.06, *P* = 0.81) between interviewed suicide attempters and those who were not interviewed.

Controls were matched to case on age (±3 years), gender, and residence in the same village. Village doctors, who provide healthcare and know villagers very well in rural China, were requested to identify potential controls for each suicide attempter. One control was randomly selected if 2 or more potential controls were available.

Interviews were conducted at least one month after the suicide attempt with a median interval of 5 months. A structured questionnaire was used to collect data by interviewing cases and controls. All interviewers received rigorous training in interviewing skills and data collection and passed the Collaborative Institutional Training Initiative (CITI) course. The CITI program (https://www.citiprogram.org/) is an international online education resource for human subjects protection and the responsible conduct of research [15]. Interviews were conducted in participants’ houses or village clinics and were tape recorded to document the entire interview process. Interview time averaged 1.5 hours. Before the interview, the interviewers explained the objectives of the study and obtained interviewees’ written consent. All participants volunteered to complete the interview without incentive.

### Instruments

Demographic information included age, gender, education level, marital status, occupation (farmer or others), family economic status, religion, party/league membership, perceived health status, physical illness, family history of completed/attempted suicide, and personal authority in the family, etc. Personal authority in the family is defined as one’s position and importance within his/her family and determined by asking interviewees the question “How do you think of your personal authority in your family?”

The Structured Clinical Interview for DSM-IV (SCID) was used by psychiatrist to diagnose mental disorders [16]. The Chinese version of the SCID has been widely used in clinical and epidemiological studies in China [9, 17].

NLEs were assessed by the Life Event Scale (LES) [18]. The LES is a modified version of Paykel’s Interview for Recent Life Events [18, 19]. The LES consists of 64 life events in five categories: marital events (14 items), family events (18 items), work-study events (10 items), health events (13 items), and legal and others events (9 items) (see S1 Information). Each event was self-reported for occurrence (1 = “yes”, 0 = “no”), time of occurrence (within 1 month, 1–3 months, 3–6 months, 6–12months prior to attempted suicide or interview for controls), frequency, nature (1 = “good”, 2 = “bad”), degree of influence on mental status and the duration of influence. NLEs were defined as those bad events by nature based on subject’s response. This scale has good reliability and validity for suicidal studies in China [20].

### Statistical analysis methods

Statistical Program for Social Sciences (SPSS, version 16.0), was used for statistical analysis. Quantitative data was described in mean ± SD, and categorical data in proportion [n (%)]. The means of two samples were compared using *t* test, and chi-square analysis was used to compare categorical data. Univariate and multivariate conditional logistic regression models were used to examine the associations between NLEs and attempted suicide. Factors that were significantly associated with attempted suicide in the univariate analyses or that have been previously reported to be associated with suicidal behavior were selected as covariates in multivariate regression analyses, including mental disorder [21], physical illness [22], perceived health status [23], education level [21], occupation [24], economic status [25], membership of the Communist Party/League [4], family history of suicide [24], and personal authority in the family.

This work follows the guideline of the strengthening the reporting of observational studies in epidemiology (STROBE) statement (see S2 Information) [26].

### Ethical Statement

The protocol obtained approval from the Institutional Review Board of the School of Public Health, Shandong University. Informed consent forms were signed by subjects before the interview; for minors who have incompetence on judgment (subjects under 18 years old), written consents from parents and themselves were obtained according to ethical guide [27, 28].

## Results

### (1) Characteristics of suicidal behavior

Of the 409 suicide attempters, 132 (32.3%) were male, and 277 (67.7%) were female, and mean age was 43.90 (SD = 13.32). Most suicide attempts happened at home (354 cases, 86.6%) and pesticide was the most commonly used method (344 cases, 84.1%). 331 (80.9%) suicide attempters made no suicide plan or preparation and 373 (91.2%) made no post mortem arrangements. In addition, 36 (8.8%) suicide attempters had previously attempted suicide and had a family history of completed/attempted suicide.

### (2) Comparison of demographic characteristics between suicide attempters and controls

Suicide attempters and controls did not differ in average age, marital status, living status (alone vs. cohabiting), and religious belief. Suicide attempters were significantly more likely than controls to have a lower educational level, be a farmer, have a physical illness or mental disorder, be a non-member of the Communist Party/League, and have a family history of completed/attempted suicide. Significant differences were also found in personal authority in the family, economic status, perceived health status between the two groups. See Table 1.

**Table 1:** Demographic characteristics in suicide attempters (N = 409) and controls (N = 409).

**Variables**	**Suicide attempters**	**Controls**	***P***
	**Mean (SD)/ n (%)**	**Mean (SD)/ n (%)**	
Age	43.90±13.32	43.74±13.26	0.153
Gender			1.000
Male	132 (32.3)	132 (32.3)	
Female	277 (67.7)	277 (67.7)	
Education level			<0.001
Lower than high school	383 (93.6)	329 (80.4)	
High school or higher	26 (6.4)	80 (19.6)	
Farmer			<0.001
Yes	299 (73.1)	243 (59.4)	
No	110 (26.9)	166 (40.6)	
Marital status			0.215
Married	349 (85.3)	361 (88.3)	
Others	60 (14.7)	48 (11.7)	
Live alone			0.374
Yes	19 (4.6)	14 (3.4)	
No	390 (95.4)	395 (96.6)	
Personal authority in family			<0.001
High	279 (68.2)	310 (75.8)	
Ordinary	92 (22.5)	86 (21.0)	
Low	38 (9.3)	13 (3.2)	
Economic status			<0.001
Poor	132 (32.2)	66 (16.1)	
Fair	233 (57.0)	267 (65.3)	
Good	44 (10.8)	76 (18.6)	
Perceived health status			<0.001
Poor	98 (24.0)	39 (9.5)	
Fair	89 (21.8)	75 (18.3)	
Good	222 (54.3)	295 (72.1)	
Physical illness			0.006
Yes	130 (31.8)	95 (23.2)	
No	279 (68.2)	314 (76.8)	
Mental disorder			<0.001
Yes	132 (32.3)	20 (4.9)	
No	277 (67.7)	389 (95.1)	
Family history of completed suicide/attempted suicide			0.027
Yes	36 (8.8)	20 (4.9)	
No	373 (91.2)	389 (95.1)	
Religious faith			0.542
Yes	14 (3.4)	11 (2.7)	
No	395 (96.6)	398 (97.3)	
Party/League member			0.007
Yes	51 (12.5)	79 (19.3)	
No	358 (87.5)	330 (80.7)	

### (3) Individual NLEs and attempted suicide

A series of conditional logistic regressions were conducted to examine the association between each NLE in the last year and attempted suicide. Of the 64 NLEs, 15 events were significantly related to increased risk of attempted suicide. As shown in Table 2, quarrelling with spouse had the largest odds ratio (*OR* = 42.67, *95% CI*: 13.58–134.05) and highest incidence in suicide attempters (31.5%). Other three NLEs with an *OR* value larger than 10 were: quarrelling with other family members (*OR* = 20.33, *95% CI*: 6.38–64.80), fighting with spouse (*OR* = 14.50, *95% CI*: 3.46–60.77), and fighting with other family members (*OR* = 12.00, *95% CI*: 1.56–92.29). We then conducted a multivariate logistic regression model including all NLEs and demographic factors. As shown in Table 2, five NLEs were still significantly associated with increased risk of attempted suicide: quarrelling with spouse (*OR* = 178.08, *95% CI*: 29.74–1066.46), quarrelling with other family members (*OR* = 113.41, *95% CI*:12.50–1028.87), serious illness (*OR* = 9.16, *95% CI*: 2.70–31.07), conflicting with friends or neighbors (*OR* = 7.09, *95% CI*: 1.82–27.68), and family financial difficulty (*OR* = 4.11, *95% CI*: 1.48–11.40).

**Table 2:** NLEs related to attempted suicide and adjusted risk of attempted suicide by conditional logistic regression model.

**NLE**	**Suicide attempters**	**Controls**	**OR (95%CI)**	Adjusted OR* (95%CI)
	**n (%)**	**n (%)**		
Love failure	9 (2.2%)	1 (0.2)	9.00 (1.14–71.04)	—
Spouse unfaithful	16 (3.9)	3 (0.7)	5.33 (1.55–18.30)	—
Quarrelling with spouse	129 (31.5)	4 (1.0)	42.67 (13.58–134.05)	178.08 (29.74–1066.46)
Fighting with spouse	29 (7.1)	2 (0.5)	14.50 (3.46–60.77)	—
Quarrelling with other family members	63 (15.4)	5 (1.2)	20.33 (6.38–64.80)	113.41 (12.50–1028.87)
Fighting with other family members	12 (2.9)	1 (0.2)	12.00 (1.56–92.29)	—
Major loss in property	13 (3.2)	3 (0.7)	4.33 (1.24–15.21)	—
Family financial difficulty	54 (13.2)	13 (3.2)	5.10 (2.59–10.05)	4.11 (1.48–11.40)
In discord with spouse’s mother	15 (3.7)	3 (0.7)	7.00 (1.59–30.80)	—
Problems in disciplining children	19 (4.6)	8 (2.0)	2.38 (1.04–5.43)	—
Serious illness	37 (9.0)	11 (2.7)	4.25 (1.97–9.18)	9.156 (2.698–31.065)
Hospitalized	39 (9.5)	15 (3.7)	2.85 (1.51–5.35)	—
Conflicting with friends or neighbors	25 (6.1)	4 (1.0)	6.25 (2.18–17.96)	7.09 (1.82–27.68)
Face-loss	18 (4.4)	6 (1.5)	3.00 (1.19–7.56)	—
Daily life out of routine	33 (8.1)	13 (3.2)	2.54 (1.34–4.82)	—

*Adjusted for education level, occupation, economic status, personal authority in the family, family history of completed suicide/attempted suicide, membership of the Communist Party/League, perceived health status, physical illness, mental disorders and all other NLE items. Only NLEs of significance were listed.

### (4) Numbers of NLEs and attempted suicide

Suicide attempters experienced more NLEs in the last year than controls (*P*<0.01), with a mean number of 1.76±1.42 in suicide attempters, and 0.56±1.07 in controls. The logistic regression model demonstrated a significant dose-response relationship between the number of NLEs in the last year and risk of attempted suicide (Cochran-Armitage trend test, *Z* = 14.28, *P*<0.01). The dose-response relationship remained significant even after controlling for demographic factors and mental disorders. Compared to those who experienced no NLEs, individuals who reported 1 or 2 NLEs were 7 times more likely to attempt suicide, the risk increased to 11 times for those who experienced 3 or more NLEs. See Table 3.

**Table 3:** Number of NLEs within the last year and risk of attempted suicide.

**Number of NLEs**	**Suicide attempters**	**Controls**	**OR (95%CI)**	Adjusted OR* (95%CI)
	**n (%)**	**n (%)**		
0	69 (6.9)	272 (66.5)	1.00	1.00
1–2	240 (58.7)	119 (29.1)	7.38 (4.81–11.30)	6.75 (4.27–10.68)
≥3	100 (24.4)	18 (4.4)	19.39 (9.88–38.06)	11.00 (5.18–23.35)

^*^Adjusted for education level, occupation, economic status, personal authority in the family, family history of completed suicide/attempted suicide, membership of the Communist Party/League, perceived health status, physical illness, and mental disorders.

### (5) Timing of NLEs and attempted suicide

The incidences of NLEs between cases and controls across 4 time periods prior to suicide attempt or prior to interview in controls are presented in Table 4. Within the past year, 83.1% of suicide attempters experienced at least 1 NLE, compared to 33.5% in controls (*χ^2^* = 207.240, *P* <0.01). A total of 118 (28.9%) suicide attempters and 30 (7.3%) controls experienced NLEs across 2 or more time periods.

**Table 4:** NLEs across 4 time periods and attempted suicide.

**NLEs**	**Suicide attempters**	**Controls**	**OR (95%CI)**	Adjusted OR* (95%CI)
	**n (%)**	**n (%)**		
Last 1 month				
No	203 (49.6)	380 (92.9)	1.00	1.00
Yes	206 (50.4)	29 (7.1)	18.70 (9.90–35.33)	21.74 (10.32–45.79)
Last 1–3 months				
No	364 (89.0)	376 (91.9)	1.00	—
Yes	45 (11.0)	33 (8.1)	1.40 (0.88–2.24)	—
Last 3–6 months				
No	367 (89.7)	375 (91.7)	1.00	—
Yes	42 (10.3)	34 (8.3)	1.27 (0.79–2.04)	—
Last 6–12 months				
No	230 (56.2)	331 (80.9)	1.00	1.00
Yes	179 (43.8)	78 (19.1)	3.41 (2.41–4.80)	3.27 (1.99–5.38)

^*^Adjusted for education level, occupation, economic status, personal authority in the family, family history of completed suicide/attempted suicide, membership of the Communist Party/League, perceived health status, physical illness, and mental disorders.

We examined the relationship between NLEs across four time periods and attempted suicide. Logistic regression models indicated that NLEs within the last month and 6–12 months prior to suicide attempts were significantly associated with attempted suicide. The associations remained significant even after adjusting for demographic factors and mental disorders. The risk of suicide attempts was much higher in those individuals who experienced NLEs in the last month prior to suicide than those who experienced NLEs in the last 6–12 months. The risk of suicide attempts was more than 20 times higher in individuals who experienced NLEs in the last month compared to those without NLEs. See Table 4.

### (6) Other risk factors associated with attempted suicide

Multivariate logistic regression found that the following factors were independently associated with attempted suicide after adjusting for NLEs and other demographic factors. They were occupation of farmer vs. non-farmers (*OR* = 1.88, *95% CI*: 1.09–3.24), low education level (*OR* = 2.37, *95% CI*: 1.10–5.11), low personal authority in the family (*OR* = 3.58, *95% CI*: 1.34–9.52), and mental disorders (*OR* = 8.39, *95% CI*: 4.11–17.12).

## Discussion

While a number of studies have found the association between NLEs and suicidal behavior [7, 18, 29, 30], this current study represents one of the largest studies to examine the independent association between NLEs and attempted suicide while adjusting for the potential confounding effects of demographic factors and mental disorders in rural China. The major findings from this study are: (1) there was a dose-response relationship between NLEs in the preceding year and attempted suicide; (2) NLEs within the last month and the previous 6–12 months were associated with attempted suicide; (3) Interpersonal conflicts with spouse, other family members and friends or neighbors, serious physical illness, and family financial difficulties were the major NLEs associated with increased risk of attempted suicide; and (4) occupation of farmer, low education level, low personal authority in the family, and mental disorders were also independently associated with attempted suicide in rural China.

### Frequency of NLEs and attempted suicide

In this study, NLEs had higher incidence in suicide attempters than in controls across all four time periods. We also found a dose-response relationship between number of NLEs and attempted suicide, consistent with previous studies [8, 31–33]. As a risk factor, NLEs may directly cause attempted suicide as a solution or avoidance of the difficulties and stress derived from them, or indirectly facilitate the behavior by generating and aggravating psychiatric symptoms [34, 35]. NLEs may also influence the stress system by altering biological stress systems (hormones and neurotransmitters), and the subsequent imbalance in turn may lead to suicidal behavior [36].

### Type of NLEs and attempted suicide

Analyses of NLEs indicated that 5 NLEs were independently associated with attempted suicide after adjusting for demographic factors, mental disorders, and other NLEs. Of the 5 NLEs, 3 are related to interpersonal conflicts (quarrelling with spouse, quarrelling with other family members, and conflicting with friends or neighbors) and 2 are chronic events including family financial difficulties and serious illness. Similar events have been reported to be associated with suicidal behavior in Western populations [37–41]. Family financial difficulties and serious illness may have long-term impact on suicidal behavior and the interpersonal events may play a trigger/precipitating role in suicide attempt. Therefore, psychosocial intervention with a focus on acute and chronic stress management and coping with interpersonal conflicts may be important steps in reducing suicidal risk in rural China.

### Time of NLEs and attempted suicide

We found that NLEs in the last month and 6–12 months were significantly associated with increased risk of attempted suicide. NLEs may occur intermittently or frequently, or even exist for a long time, and may be aggravated by continuation [42]. Several studies found that suicidal individuals experienced more frequent NLEs on the day of suicidal event, in the previous week, and in the previous 3 or 6 months [29, 43, 44]. In the current study, we found that the incidences of NLEs within the last month, 1–3 months, 3–6 months, and 6–12 months prior to suicide attempts were higher in suicide attempters than in controls. Furthermore, we found that NLEs in the last month and 6–12 months intervals, rather than in the last 1–3 months and 3–6 months intervals, were significantly associated with increased risk of suicide attempts. NLEs within the last month are usually acute and stressful, like interpersonal conflicts (quarrelling/fighting with spouse or other family members). These acute NLEs may play a precipitating role in suicide attempts, especially for impulsive attempters. NLEs within the last 6–12 months may be major and chronic events, such as family financial difficulties, chronic and serious illness, and death of close family members and may have chronic and sustained impact on suicidal behavior [9, 42].

The incidences of NLEs for suicide attempters were much lower in the last 1–3 months and 3–6 months than in the last month and 6–12 months. The incidences of NLEs in the control group were relatively consistent across the 4 time periods. This may explain why NLEs in the 2 time periods were not significantly associated with suicide attempts.

The differences of NLEs between suicide attempters and controls in the last 6–12 months have 2 possible explanations. Suicide attempters experienced more chronic stressful NLEs in the 6–12 months, which had long-term effects on suicidal behavior. On the other hand, the difference may be due to recalling bias that suicide attempters were more likely to recall all NLEs happened 6 months prior to suicide attempts as events happened in the period of 6–12 months because our study did not ask the events that happened 1 year before. It would be interesting and important to examine the NLEs happened 1 year before the attempt in the future studies.

### Other risk factors

Consistent with previous studies, we found that low education level [21, 45] and mental disorders [21, 46] were significantly associated with increased risk for suicide attempts. We also found that low personal authority in the family was independently associated with attempted suicide. Low personal authority in the family means holding a dominated and governed position in the family. Individuals with a low authority in the family are often disrespected and some are even abused emotionally, verbally, and physically by other family members. These individuals are more likely to have low self-esteem, hopelessness, and mental health problems, all of which may increase risk for suicide attempts [46–48]. It should be noted that although mental disorder was a significant risk factor for attempted suicide, only 32.3% of suicide attempters in this study were diagnosed with a mental disorder, which is much lower than those in western countries [44, 49]. In the current study, most suicide attempters had no preparation and made no will for suicide. Furthermore, NLEs in the last month were much more frequent in suicide attempters than in controls. Taking all these together, we conclude that most suicide attempts in rural Shandong may be an impulsive behavior, precipitated by stressful life events, especially from interpersonal conflicts.

### Limitations and strengths

Some limitations in this study should be noted. Recalling bias may be a strong limitation as reporting of past NLEs was likely to be affected by the very outcome of a suicide attempt. Suicide attempters admitted to emergency wards may represent those with more severe attempts. Thus, the entire spectrum of suicide attempts was not captured in this study. Selection of controls was under the help of village doctors, which may lead to an incomplete random sample. The high rate of lost follow-up due to incorrect contact information or migration to the city was another limitation. Although there were no significant differences in mean age and gender distribution between those interviewed and not-interviewed, the interviewed suicide attempters may not be representative of all attempters in the area. In addition, some odds ratios with large confidential intervals due to low incidences of some NLEs and small sample size maybe overestimated [50]. Furthermore, some potential confounders like personality and coping style were not assessed in the study. Finally the causal relationship between NLEs and suicide attempts may not be concluded from the retrospective study. Further studies in this subject are warranted to make sound decisions in suicide prevention in rural China.

This study also has several strengths. Shandong Province exemplifies features of China’s traditional culture and development in recent decades, and the results may be generalized to the rest of the nation. As far as we know, this study is the first of which to evaluate NLEs in several sequential time periods among a large sample of rural suicide attempters. The dose-response relationship between NLEs within 4 time periods and attempted suicide was examined. In addition, mental disorders were assessed by standardized Structured Clinical Interview for DSM-IV (SCID) and their effects were adjusted when examining the association between NLEs and suicide attempt.

In conclusion, this study represents one of the largest studies to examine the independent association between NLEs and attempted suicide while adjusting for the potential confounding effects of demographic factors and mental disorders in rural China. NLEs are significantly associated with increased risk for attempted suicide. While mental disorders are a risk factor of suicide attempts in rural China, negative life events, especially interpersonal conflicts in the last month, may play a precipitating role in suicidal behavior. Stress intervention and management of interpersonal conflicts may be important to prevent suicidal behavior in rural China.

## Supporting Information

S1 Information NLEs included in the Life Event Scale. (DOC)

S2 Information STROBE Statement—Checklist of items that should be included in reports of case-control studies. (DOC)
