# Supplementary material for: Negative Life Events and Attempted Suicide in Rural China
Source: PLoS One. 2015 Jan 22;10(1):e0116634. doi: 10.1371/journal.pone.0116634 (PMC4303417; doi:10.1371/journal.pone.0116634)
Supplement: S1 Information — (DOC) [file pone.0116634.s001.doc]

The Life Event Scale (LES) used to measure NLEs in this study includes 64 items describing common life events in five categories. The specific events are as follows:

**Martial events** (14 items): 1. Marriage 2. In love/ becomes engaged, 3. Love failure, 4. Marital separation due to argument, 5. Marital separation not due to disease, 6. Divorce, 7. Spouse unfaithful, 8. Extramarital affair, 9. Marriage/love affair interfered, 10. Breakup of extramarital affair, 11. Quarrelling with spouse, 12. Fighting with spouse, 13. Physically abused by spouse, 14. Dissatisfaction with sexual life;

**Family events** (18 items): 15. Child leaves home, 16. Child got married/parents remarried, 17. Separation/divorce of parents/child, 18. Quarrelling with other family members, 19. Fighting with other family members, 20. Major loss in property, 21. Family financial difficulty, 22. Disputing over dowry/bride-price, 23. In discord with spouse’s mother, 24. Birth of a baby, 25. Become pregnant (wife), 26. Miscarriage or stillbirth, 27. Child/ parents having a baby, 28. Infertility (husband or wife), 29. Breach the one child policy, 30.Sterilisation (husband or wife), 31. Menopause, 32. Problems in disciplining child;

**Work-study events** (10 items): 33. Bussiness failure, 34. Fired, 35. Unemployed for one month, 36. Promotion, 37. Quarrelling with boss or co-worker, 38. Dimission, 39. Dimission of spouse, 40. Decreased income, 41. Quitting school, 42. Frustrated in school/work;

**Health events** (13 items): 43. Serious illness, 44. Hospitalized, 45. Spouse hospitalized, 46. Need home nursing, 47. Serious illness of family member, 48. Injured from accident, 49. Serious illness of close friend, 50. Death of child, 51. Death of parent, 52. Death of spouse, 53. Death of sibling, 54. Death of close relative, 55. Death of close friend;

**Legal events and others** (9 items): 56. Victimized of a crime, 57. Jail sentence, 58. Legal trouble, 59. Law suit, 60. Conflicting with friends or neighbors, 61. Face-loss, 62. Threatened, 63. Sexually assaulted, 64. Daily life out of routine.
